# Transcriptome sequencing reveals the effects of circRNA on testicular development and spermatogenesis in Qianbei Ma goats

**DOI:** 10.3389/fvets.2023.1167758

**Published:** 2023-04-27

**Authors:** Wen Tang, Qiang Hou Xu, Xiang Chen, Wei Guo, Zheng Ao, Kaibin Fu, Taotao Ji, Yue Zou, Jing Jia Chen, Yuan Zhang

**Affiliations:** ^1^College of Life Science, Guizhou University, Guiyang, China; ^2^Key Laboratory of Animal Genetics, Breeding and Reproduction in the Plateau Mountainous Region, Ministry of Education, Guizhou University, Guiyang, China; ^3^Key Laboratory of Animal Genetics, Breeding and Reproduction, Guiyang, China; ^4^College of Animal Science, Guizhou University, Guiyang, China

**Keywords:** testicular development, spermatogenesis, circRNA, Qianbei Ma goats, ceRNA

## Abstract

Circular RNAs (circRNAs) play an important role in regulating the mammalian reproductive system, especially testicular development and spermatogenesis. However, their functions in testicular development and spermatogenesis in the Qianbei Ma goat, the Guizhou endemic breed are still unclear. In this study, tissue sectioning and circRNAs transcriptome analysis were conducted to compare the changes of morphology and circular RNAs gene expression profile at four different developmental stages (0Y, 0-month-old; 6Y, 6-month-old; 12Y, 12-month-old; 18Y, 18-month-old). The results showed that the circumferences and area of the seminiferous tubule gradually increased with age, and the lumen of the seminiferous tubule in the testis differentiated significantly. 12,784 circRNAs were detected from testicular tissues at four different developmental stages by RNA sequencing, and 8,140 DEcircRNAs (differentially expressed circRNAs) were found in 0Y vs. 6Y, 6Y vs. 12Y, 12Y vs. 18Y and 0Y vs. 18Y, 0Y vs. 12Y, 6Y vs. 18Y Functional enrichment analysis of the source genes showed that they were mainly enriched in testicular development and spermatogenesis. In addition, the miRNAs and mRNAs associated with DECircRNAs in 6 control groups were predicted by bioinformatics, and 81 highly expressed DECircRNAs and their associated miRNAs and mRNAs were selected to construct the ceRNA network. Through functional enrichment analysis of the target genes of circRNAs in the network, some candidate circRNAs related to testicular development and spermatogenesis were obtained. Such as *circRNA_07172, circRNA_04859*, *circRNA_07832*, *circRNA_00032* and *circRNA_07510.* These results will help to reveal the mechanism of circRNAs in testicular development and spermatogenesis, and also provide some guidance for goat reproduction.

## 1. Introduction

Reproductive traits are important economic traits in goats, and improving the fecundity of breeding rams is an important guarantee to improve the efficiency of goat breeding (1). As an important organ for the maintenance of male reproductive ability, the main biological function of testis is to produce sperm, and testicular development is a necessary process for sperm production. It is the guarantee for the establishment and maintenance of the male reproductive system (2), while, the contribution of ram testes to reproduction is as high as 50% (3). During testicular development, the genome of testicular cells is actively transcribed into mRNA and many noncoding RNAs(ncRNAs) composed of miRNAs, circRNAs, and lncRNA. circRNAs can act as a competitive endogenous RNA (ceRNA) to regulate gene expression by regulating the activity of miRNA (4), thereby participating in the biological processes of testicular development and spermatogenesis (5, 6).

CircRNAs are a prominent new class of closed-loop RNA molecules found in eukaryotes, that are produced by the alternate shearing of precursor mRNAs (7), and are involved in the regulation of mammalian development (8), growth (9), reproduction (10), and immunity (11) in animals. For example, *circPPARA* can act as an endogenous sponge to adsorb *mir-429* and *mir-200b*, thereby promoting intramuscular adipogenesis in pigs (12), and reduced expression of *circZNF609* significantly inhibits myoblast proliferation in mice *in vivo* (13). Holdt et al. (14) discovered that *circ-ANRIL* could bind to ribosome biogenesis factor 1 (PES1) to prevent ribosome formation, thereby promoting cell apoptosis and inhibiting macrophage proliferation. Liu et al. (15) identified 388 differentially expressed (DE) circRNAs in pituitary tissues of high-yield and low-yield black goats and found that *circ_0014542*, *circ_0031209* and *circ_0019448* played important roles in goat reproduction. Zhang et al. (16) detected that circRNAs in goat endometrial epithelial cells could influence the reproductive performance of goats by regulating the embryo implantation process. The above studies suggest that circRNAs play an important role in the regulation of reproduction. In goats, the research on the reproductive performance of circRNA has mainly focused on the ovary, pituitary and uterus of ewes, while its effects and mechanism testicular development and spermatogenesis of rams have not been fully clarified.

Qianbei Ma goats are a unique goat variety in the mountainous area of the Guizhou Plateau. It is a large size, strong physique, compact structure, in meat, wool and dual-purpose goat. It has many advantages, such as high fertility, strong disease resistance, strong adaptability and stable genetic performance. It is an ideal animal for studying testicular development in highland meat goats (17). Based on the importance of the testis to the reproduction of goats, the testis of Qianbei Ma goats at four developmental stages (0Y, 6Y, 12Y, and 18Y) were selected for histomorphology and transcriptomic sequencing (NA-SEQ) analysis to explore the testis developmental rules. Through DEcircRNAs analysis, circRNAs regulation network construction s, and functional enrichment analysis the key circRNAs related to testis development and spermatogenesis at different developmental stages were identified. This study aims to reveal the underlying mechanism of circRNA regulation of testicular development and spermatogenesis, and provide theoretical basis and data support for improving the reproductive performance of rams.

## 2. Materials and methods

### 2.1. Collection of animal samples headings

Twenty healthy Qianbei Ma goats were randomly selected from the same flock at Fuxing Herding Co Ltd., Xishui County, Guizhou Province, China, and divided into four age groups (n = 5 for each age group): 0Y, 0-month-old (weight: 1.66 kg ± 0.5 kg); 6Y, 6-month-old (Weight: 30.30 kg ± 1.0 kg); 12Y, 12-month-old (Weight: 43.32 kg ± 1.5 kg); 18Y, 18-month-old (Weight: 47.53 kg ± 2.0 kg). After anesthesia, the left testicular tissue was castrated and collected with sterile scissors forceps immediately, and trimmed to approximately 5 mm testicular tissue fixed in 4% paraformaldehyde solution for H&E staining, with three biological replicates at each age. Testicular tissue samples were collected and washed with phosphate buffered salt solution within 20 min. Then all samples were snap frozen in liquid nitrogen and subsequently transferred to-80°C for storage for further studies.

### 2.2. Histomorphological analysis

Hematoxylin-eosin (H&E) staining: paraffin sections were stained with hematoxylin using conventional methods, restained with eosin, and sealed with neutral resin before being placed under a biological microscope for observation and photographed. The images were scanned and saved using CaseViewer 2.4 software, followed by physicochemical index analysis using Image-ProPlus 6.0 software to measure the circumference and area of the seminiferous tubules and count the number of supporting and leydig cells per unit area, using millimeter as the standard unit.

### 2.3. RNA extraction and library construction

Total RNA was isolated from testis samples using TRIzol RNA (Thermo Fisher Scientific, China) kit under aseptic conditions.Ribosomal RNA was removed from total RNA in strict accordance with the instructions for the EvoM-MLV RT Kit with gDNA Clean for qPCR (Hunan Acres Biological Company, China). RNase R was applied to remove linear RNA from total RNA (Servicebio Technology Co., Ltd., China), and the RNA was broken into short fragments by adding the breaking reagent. Using the RNA as the template, one-strand cDNA was synthesized by using random primers of six bases, and then two-strand cDNA was synthesized by the reaction system of two-strand synthesis. In the process of cDNA synthesis, DUTP was substituted for DTTP, and then different linkers were ligated. One strand containing dUTP was digested by the UNG enzyme, and only one strand of cDNA connecting different linkers was retained one strand of cDNA was purified by using the kit, and then the end was repaired. A tail was added, and the sequencing linker was connected. Then, the fragment size was selected, and PCR amplification was carried out. The library was screened by an Agilent 2,100 Bioanalyzer (Agilent Technologies, Palo Alto, CA, USA) and sequenced by an Illumina sequencer.

### 2.4. circRNA identification and differential circRNA recognition

The raw sequencing data were processed to obtain high-quality pure sequences as follows: (a) reads containing adapters were filtered by fastp (version 0.23.0); (b) sequences containing more than 10% N; (c) all sequences containing baseA were removed; and (d) low-quality sequences (q ≤ 20). TopHat2 was used to map the clean reads to a reference genome from the National Center for Biotechnology Information (NCBI)^1^ (18), and the resulting comparisons were employed for the identification of circRNAs using Find_circ software (19). The expression of circRNAs was quantified by reads per million mapped (RPM) as a normalization method, and the DEseq2R package (1.10.1) was employed to analyze the differential expression of circRNAs. *p* value <0.05 and circRNAs with a |fold change| > 2.0 were regarded as significantly differentially expressed.

### 2.5. Go, and KEGG enrichment analysis

Gene Ontology (GO) and Kyoto Encyclopedia of Genes and Genomes (KEGG) pathway analysis of differentially expressed circRNA host genes were used for the note. GOseq software was applied for GO function analysis. Kobas software^2^ was applied to test the statistical enrichment of differential gene expression in the KEGG pathway. GO terms and KEGG pathways were considered significantly when at *p* < 0.05.

### 2.6. ceRNA network construction

miRanda^3^ and circBank^4^ analysis software was used to predict circRNA-targeted relationships with microRNAs. After the intersection of the prediction results of each analysis software, the CircrNA-targeted miRNA set was obtained. TargetScan^5^ and miRDB^6^ were used to predict the targeting relationship between miRNA and mRNA. After the prediction results were intersected, the set of miRNA-targeted mRNAs was obtained. Further integrating the relationship pairs among circRNA, miRNA and mRNA, to build the ceRNA regulatory network related to testicular development, their interaction network was visualized in Cytoscape software (v3.7.1).

### 2.7. RT–qPCR validation

Ten DEcircRNAs were randomly selected for RT qPCR sequencing verification. The FirstChoice™ RLM-RACE kit (Thermo Fisher Scientific, China) was used synthesize total RNA into complementary DNA (cDNA). Primer information used in RT qPCR is listed in Table 1. CFX 96 Real Time PCR system (Bio-Rad, Foster City, CA, USA) was employed to detect the relative gene expression.

**Table 1:** RT-qPCR primer information.

Gene name	Primer sequence	Length (bp)	Tm (°C)
*circRNA_01022*	F:5′-AAGCAGATGAGTTGAGTGGAG-3′ R:5′-TGGGCTGTTAGAAAGGTATCA-3′	298	60
*circRNA_01307*	F 5′-TTTCATCCCTTACTGGTCTCA-3′ R 5′-CTGTTGGCTTCATTATTGCTG-3′	177	60
*circRNA_03967*	F 5′-AGAAGGCCCACAGAGATTTGG-3′ R 5′-TTTTTCGCTGTCTGTGAGGAGA-3′	209	60
*circRNA_01629*	F 5′-CGGCAAATCTACTCTTGGGC-3′ R 5′-TGCCAGATCCATAAAACAGAGG-3′	224	60
*circRNA_05425*	F 5′-TTTTGGGTCTCAGGCATCCTC-3′ R 5′-GTATGGGTTAGTCCCGCCAT-3′	165	53
*circRNA_02389*	F 5′-ACCCAACTGTGTCAACCGTG-3′ R 5′-TTCATCCAAAAGGTCTGCTAAG-3′	241	60
*circRNA_07638*	F 5′-TCGGAAAATCGGCTCATTCG-3′ R 5′-ATAACACCACGCTGGACTGA-3′	234	61
*circRNA_12210*	F 5′-TTTTCTCGATTGGACCTGCGA-3′ R 5′-TGCCACTTCCAGGTGTTCTA-3′	187	61
*circRNA_04566*	F 5′-ATGCAGTTTCCCTCTATCTGCT-3′ R 5′-TGGGGAAGAGAGACCTGTGGTT-3′	215	61
*circRNA_04081*	F 5′-TTAGTGACGCCAACGGCTA-3′ R 5′-CATGCAGTATGATCGCGTGG-3′	193	61
*GAPDH*	F 5′-GCCGCATCCAGCCCC-3′ R 5′-TGGAAATGTGTGGAGGTCGG-3′	112	60

The reaction system was 20 μL: 2× RealStar Green Fast Mixture 10 μL, Primer Sense (10 pmol/μL) 1 μL, Primer Anti-sense (10 pmol/μL) 1 μL, cDNA (ng/μL) 2 μL, ddH_2_ O 6 μL. The reaction conditions were: 95°C for 2 min, 95°C for 15 s, annealing temperature (see Table 1 for details) for 30 s, and 72°C for 30 s. Solubility curve analysis was performed after 40 cycles, with a rate of 0.5°C rise every 5 s from 65°C to 95°C. Three biological replicates were set up. The specificity of the PCRs was determined by a single peak on the melting curve. Gene expression levels and normalized by GAPDH, and relative gene expression levels were calculated using the 2^−ΔΔCt^ method. PCR products were confirmed by 1.5% agarose gel electrophoresis, and Sanger sequencing was then performed with PCR products to verify circRNA cyclization loci.

### 2.8. Statistics

SPSS 21.0 software (SPSS, Inc., Chicago, IL, USA) was used for statistical analysis (20). All values are expressed as the mean ± SEM. The difference between the two groups was expressed with *p < 0.05 and **p < 0.01 means that each experiment should be repeated at least 3 times.

## 3. Results

### 3.1. Histomorphological analysis of the testes at different ages in Qianbei Ma goats

**Figure 1:**
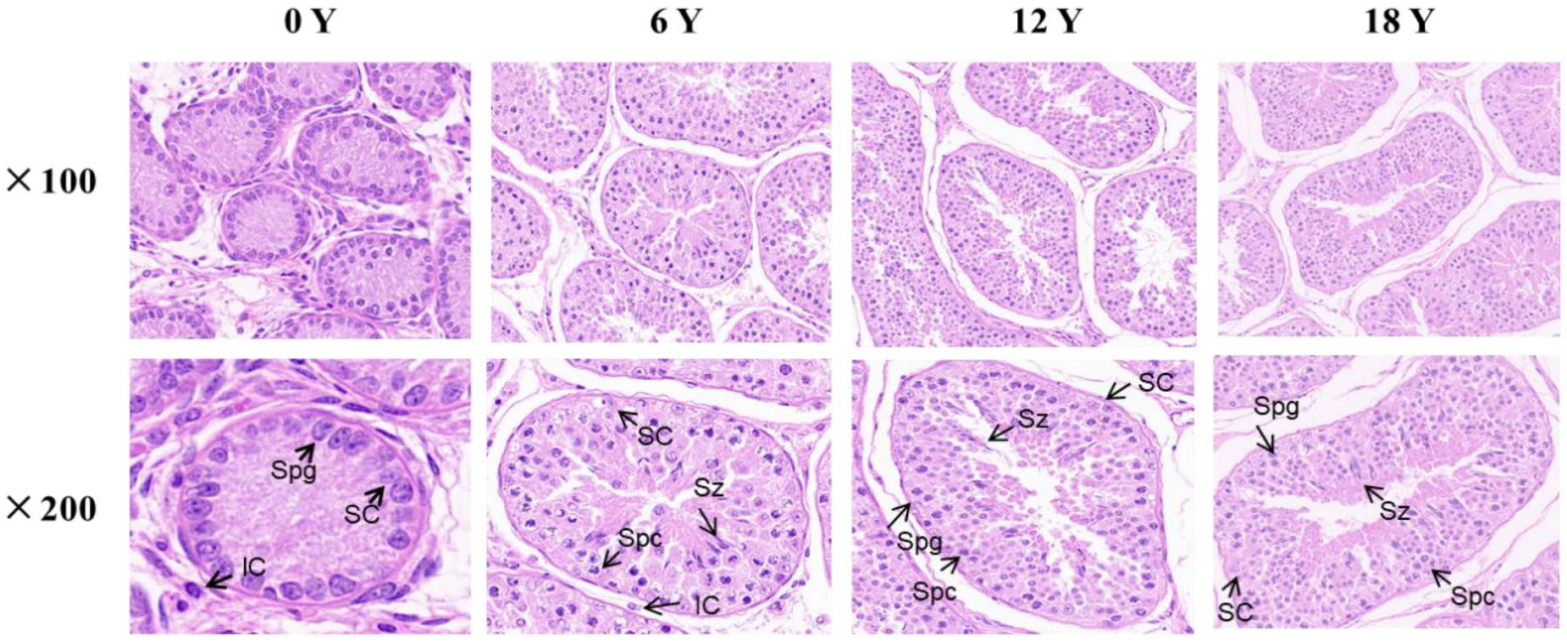
HE staining of testicular tissue from rams at different months of age. SC: supporting cells. Spg: spermatogonia. Spc: spermatocytes. LC: Leydig cells. Sz: spermatozoa.

The testis tissue is composed of seminiferous tubules and testis stroma (21). As showed in Figure 1, only Sertoli cells and spermatogonia appear at 0 months in goats, and spermatozoa appear in the seminiferous tubule lumen at 6 months, indicating the onset of puberty in Qianbei Ma goats. Compared with 0-month-old goats, the lumen of convoluted seminiferous tubules in the testis differentiated significantly at the age of 6, 12, and 18 months, and the volume of Sertoli cells increased significantly. Compared with 6 months goats, the number of layers of spermatogenic cells and the number of sperm in the lumen of the testis increased significantly in 12-month-old and 18-month-old goats. Statistical analysis of seminiferous tubules and related cells in testis also showed that, with increasing age, the circumference and area of seminiferous tubules gradually increased, and the number of spermatocytes, Sertoli cells and sperm in testis gradually increased, while the number of leydig cells gradually decreased. However, the number of Sertoli cells and Leydig cells reached the limit in 6 months goats, and the number of spermatogonia was almost the same from 12 months to 18 months, and decreased at 18 months (see Table 2).

**Table 2:** Histomorphological and physicochemical indices of the testis.

Incdex/Time	Different months			
Items	0	6	12	18
Seminiferous tubule area	0.0033 ± 0.0003^a^	0.0327 ± 0.0062^b^	0.0474 ± 0.0059^c^	0.0484 ± 0.0074^c^
Circumferences of seminiferous	0.2151 ± 0.01369^a^	0.6747 ± 0.0562^b^	0.8003 ± 0.0518^c^	0.8137 ± 0.0639^c^
Sertolicell	3.3333 ± 1.4974^a^	17 ± 4.6122^b^	13.6667 ± 4.2283^b^	15.5 ± 5.6165^b^
Leydigcell	260.1667 ± 55.3728^a^	47.1667 ± 15.8391^b^	48.1667 ± 15.4674^b^	47.6667 ± 16.3447^b^
spermatogonia	25.0833 ± 3.7769^a^	24.4167 ± 8.95908^a^	25.5833 ± 6.8551^a^	18.25 ± 4.6147B^b^
spermatocyte	0^a^	109 ± 42.3040^b^	126.416 ± 63.4843^b^	150.333 ± 52.3073^b^
spermatozoon	0^a^	44.6667 ± 10.11599^b^	94 ± 5.2915^c^	101 ± 14.42221^c^

Different lowercase letters indicate significant differences between different months of age (*p* < 0.05).

### 3.2. Transcriptome sequencing of the circRNA in testis of the Qianbei Ma goats

**Figure 2:**
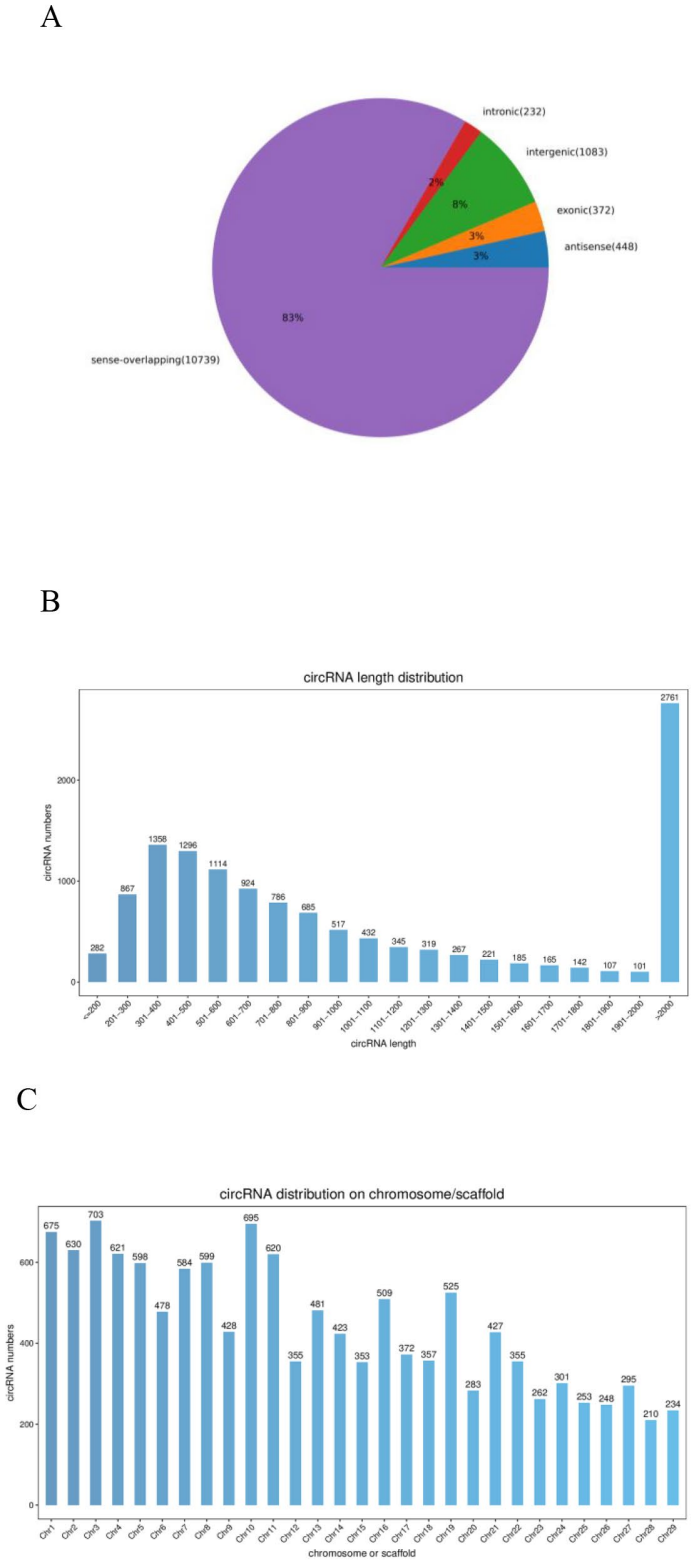
Qianbei Ma Goat testicular circRNAs. **(A)** CircRNAs from distinct genomic sites. **(B)** Length distributions of the known circRNAs. **(C)** Chromosome distribution map of circRNAs.

After removing low-quality reads, 19,706,582 clean reads were obtained (480, 343, 016 from the 0Y group; 496, 921, 424 from the 6Y group; 509, 827, 848 from the 12Y group; 479, 974, 294 from the 18Y group) (Table 3). In the 0Y, 6Y, 12Y and 18Y samples, 98.34, 97.97, 97.42 and 98.4% of the reads were located in the reference genome, and the base rates of Q30 were 91.7, 91.58, 90.72 and 91.54%, respectively. A total of 12,874 circRNAs were identified and designated by numbering from *circRNA_00001* to *circRNA_12,874*. Based on their position in the goat genome, 83% of circRNAs were found to be sense overlapping, while the other 17% were intergenic, exon, antisense and intronic (Figure 2A). The majority of circRNAs were distributed within 2000 nt in length, but the most circRNAs clustered at more than 2000 nt. (Figure 2B). These circRNAs were distributed on 29 autosomes, with higher distribution on chromosomes 1, 3 and 10 (Figure 2C).

**Table 3:** CircRNA sequencing data.

Sample^a^	RawReads^b^	RawBases^c^	CleanReads^d^	CleanBases^e^	ValidBases^f^	Q30^g^	GC^h^
12Y1	100.68 M	15.10G	99.41 M	14.76G	97.77%	92.33%	49.20%
12Y2	103.86 M	15.58G	101.68 M	15.06G	96.64%	91.33%	50.55%
12Y3	106.43 M	15.96G	104.63 M	15.53G	97.27%	92.04%	50.25%
12Y4	102.32 M	15.35G	100.64 M	14.94G	97.31%	91.71%	50.33%
12Y5	105.10 M	15.77G	103.47 M	15.35G	97.34%	91.09%	50.12%
6Y1	83.39 M	12.51G	81.09 M	11.89G	95.07%	91.40%	49.52%
6Y2	102.76 M	15.41G	100.70 M	14.95G	96.97%	91.45%	50.37%
6Y3	112.42 M	16.86G	110.05 M	16.28G	96.54%	91.63%	50.74%
6Y4	105.33 M	15.80G	103.50 M	15.37G	97.28%	91.79%	50.69%
6Y5	103.22 M	15.48G	101.59 M	15.09G	97.46%	91.63%	49.73%
OY1	99.63 M	14.94G	96.89 M	14.35G	96.02%	91.26%	49.45%
OY2	108.66 M	16.30G	106.38 M	15.76G	96.69%	91.61%	48.74%
OY3	98.99 M	14.85G	97.14 M	14.45G	97.31%	89.46%	47.59%
OY4	101.38 M	15.21G	98.97 M	14.65G	96.35%	90.91%	47.48%
OY5	84.15 M	12.62G	80.98 M	11.93G	94.49%	90.36%	48.66%
18Y1	107.00 M	16.05G	105.38 M	15.61G	97.27%	91.78%	49.50%
18Y2	95.91 M	14.39G	94.75 M	14.08G	97.89%	91.51%	49.14%
18Y3	84.71 M	12.71G	83.35 M	12.34G	97.11%	91.32%	49.60%
18Y4	99.78 M	14.97G	98.26 M	14.58G	97.43%	91.75%	49.92%
18Y5	100.40 M	15.06G	98.23 M	14.55G	96.62%	91.36%	50.36%

a 0Y1-5 represent 0-month-old. 6Y1-5 represent 6-month-old. 12Y1-5 represent 12-month-old. 18Y1-5 represent 18-month-old.

b The number of reads in the original data.

c The number of bases in the original data.

d The number of reads after filtering the original data.

e The number of bases after filtering the original data.

f The proportion of effective data.

g The proportion of effective data.

h The percentage of G and C in the four bases in clean reads.

### 3.3. Differential expression analysis of circRNAs in the testis of Qianbei Ma goats

**Figure 3:**
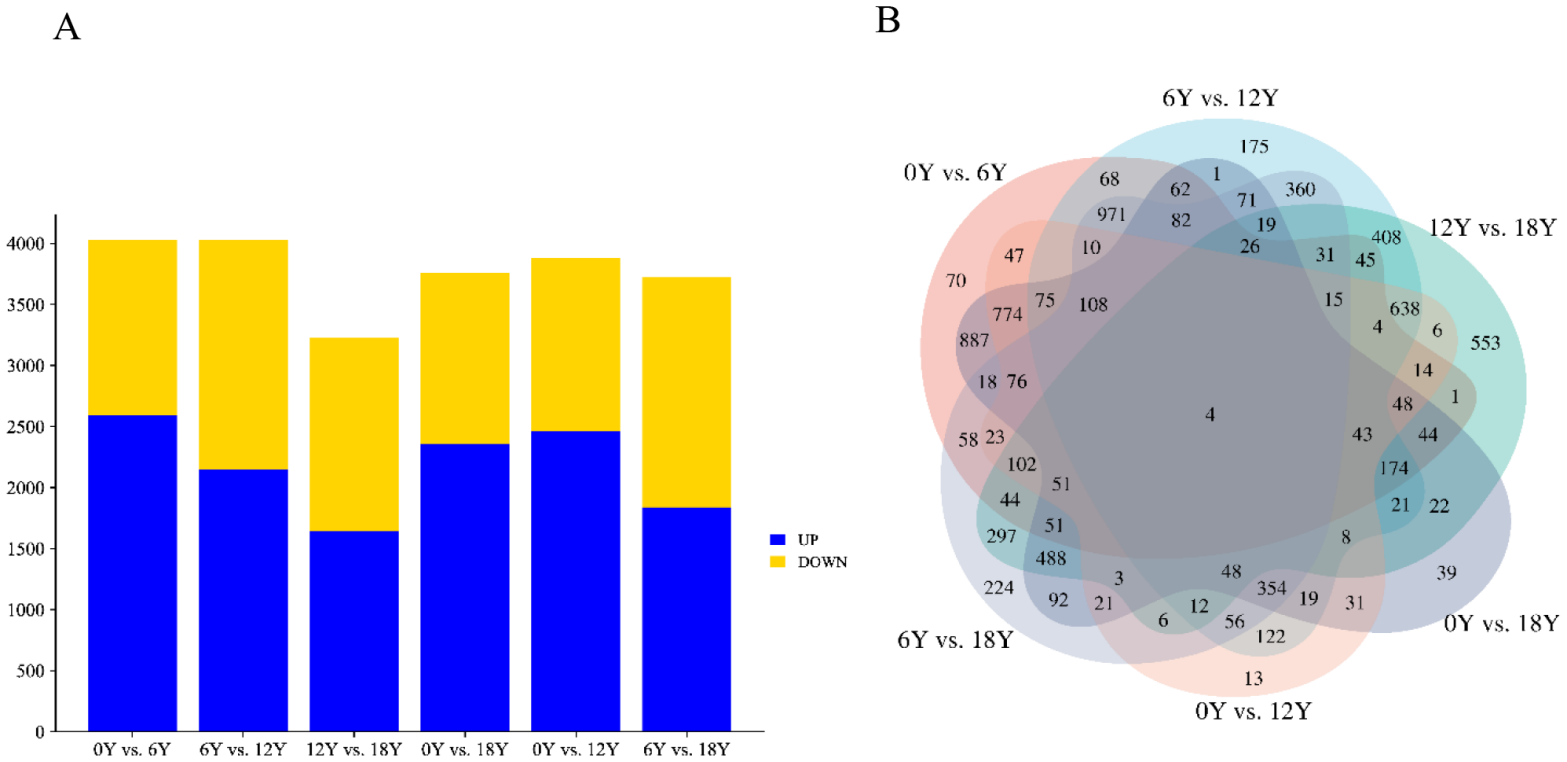
Summary of differential expression analysis of annotated circRNAs. **(A)** Number of DEcircRNAs in different control groups. **(B)** Wayne diagram of DEcircRNAs in different control groups.

**Figure 4:**
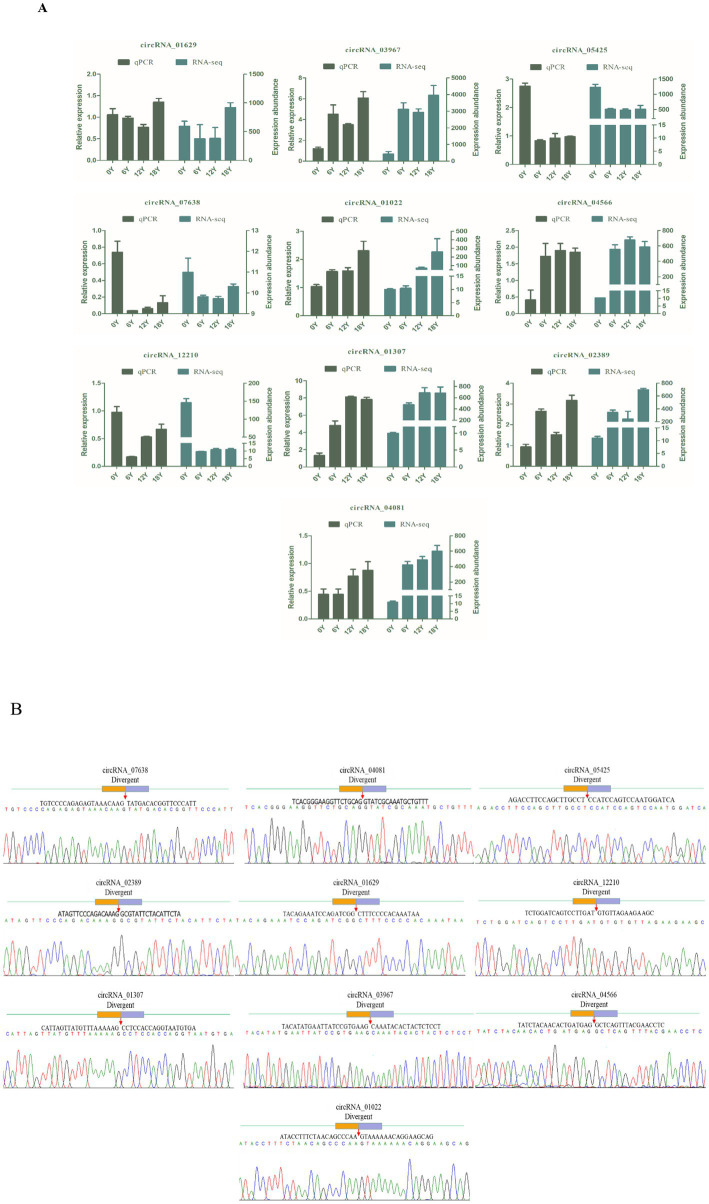
circRNA RT–qPCR validation and Sanger sequencing. **(A)** Comparison of qPCR and RNA-seq results for 10 circRNAs. **(B)** Circular junctions were confirmed by Sanger sequencing.

In the circRNA differential expression analysis, we found 8,140 differentially expressed circrnas in the six comparison groups. (Figure 3A). Specifically, 0Y vs. 6Y had 4,026 differentially expressed circRNAs (up: 2596, down: 1430), 6Y vs. 12Y had 4,030 (up: 2152, down: 1878), 12Y vs. 18Y had 3,226 (up: 1645, down: 1581), 0Y vs. 18Y had 3,760 (up: 2359, down: 1401), 0Y vs. 12Y had 3,878 (up: 2465, down: 1413), and 6Y vs. 18Y had 3,721 (up: 1839 down: 1882), of which 4 circRNAs were coexpressed in four age groups (Figure 3B). Ten DEcircRNAs were then randomly selected to validate the sequencing results by RT–qPCR, and the expression trends of the detected circRNAs were consistent with the RNA-seq sequencing results (Figure 4A). The PCR products were subjected to Sanger sequencing, verifying that the selected circRNA had the correct cyclization linker site (Figure 4B). These results indicated that the circRNA sequencing data were reliable, and could be used for further analysis.

### 3.4. Functional enrichment and annotation analysis of host genes of DEcircRNAs

Analysis of the origin of the DEcircRNAs we obtained showed that in the 0Y vs. 6Y comparison, 3,729 of the 4,026 identified were from 2,221 source genes, with the remaining 297 circRNAs from intergenic regions. In the 6Y vs. 12Y group, 3,725 of the 4,030 DEcircRNAs identified were from 2,168 source genes and the remaining 305 circRNAs were from intergenic. In the 12Y vs. 18Y group, 2,963 of the 3,226 DEcircRNAs identified were from 1852 source genes and the remaining 263 circRNAs were from intergenic regions. In the 0Y vs. 18Y group, 3,760 of the 3,760 of the 3,497 DEcircRNAs identified were from 2040 source genes, with the remaining 263 circRNAs from intergenic regions. In the 0Y vs. 12Y group, 3601of the 3,878 DEcircRNAs identified were from 1885 source genes and the remaining 277 circRNAs were from intergenic regions. In the 6Y vs. 18Y group, 3428of the 3,721 DEcircRNAs identified were from 2034 source genes and the remaining 293 circRNAs were from intergenic regions.

**Figure 5:**
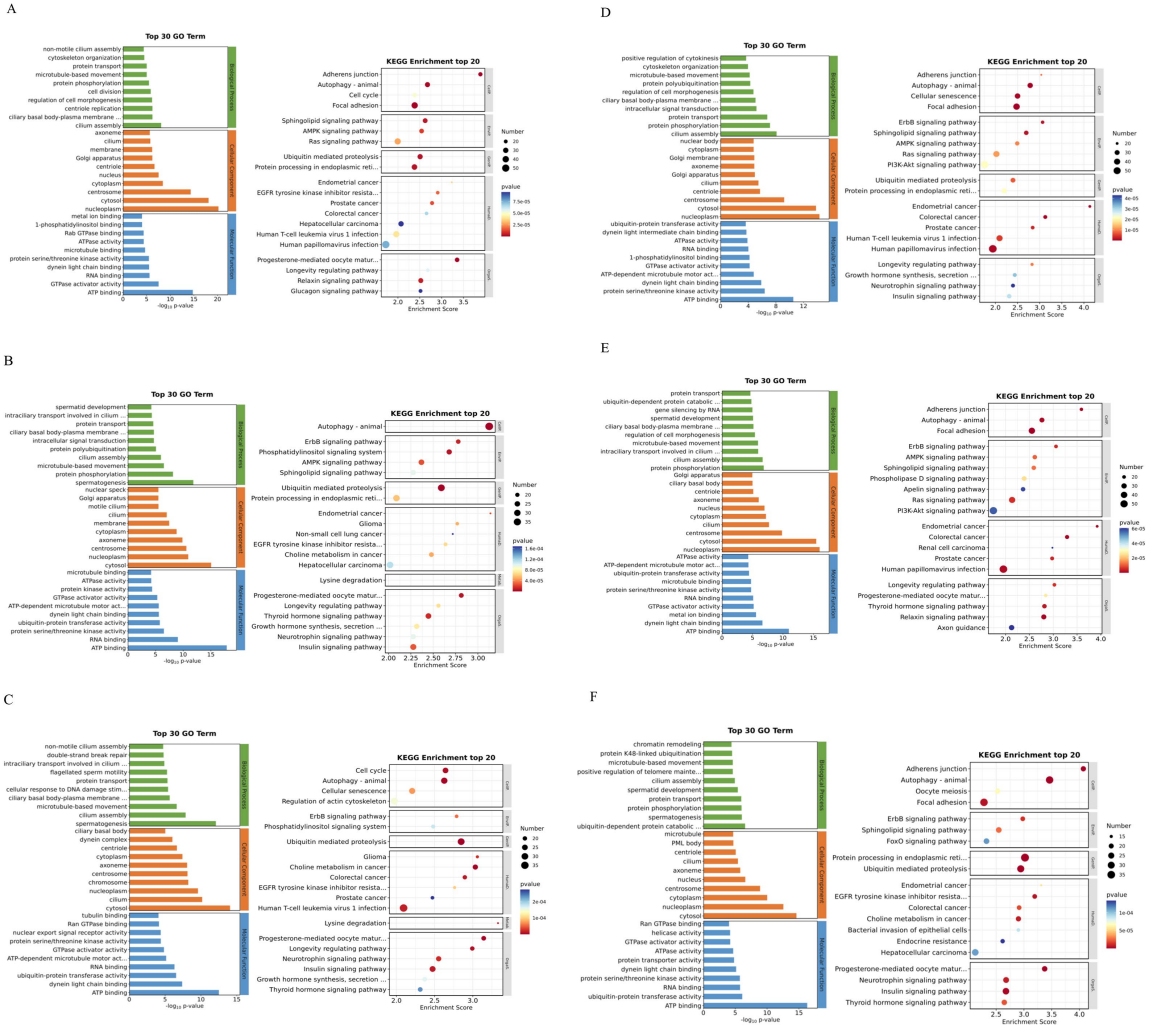
GO annotation and KEGG enrichment analysis of differential circRNA source genes in different control groups. **(A)** 0Y *vs.* 6Y, **(B)** 6Y *vs.* 12Y, **(C)** 12Y *vs.* 18Y, **(D)** 0Y *vs.* 18Y, **(E)** 0Y *vs.* 12Y, and **(F)** 6Y *vs.* 18Y; 0Y, 6Y, 12Y and 18Y refer to October, June, December and August, respectively.

The results of GO enrichment analysis showed that 0Y vs. 6Y, 0Y vs. 12Y and 0Y vs. 18Y were mainly involved in cilia assembly (GO:0060271), gonad development (GO:0008406), protein transport (GO:0015031) and regulation of cell morphogenesis (GO:0022604). In terms of cellular components, they mainly included centrosome (GO: 0005813), cytoplasm (GO: 0005737) and Golgi apparatus (GO: 0005794). In terms of molecular function, the main focus was on ATP binding (GO: 0005524), and microtubule binding (GO: 0008017) (Figures 5A,D,E). The source genes of the DEcircRNAs 6Y vs. 12Y, 6Y vs. 18Y and 12Y vs. 18Y were dominated by spermatogenesis (GO: 0007283), protein phosphorylation (GO: 0006468), cilia assembly (GO: 0060271), spermatocyte development (GO: 0007286), which was very similar to 0Y vs. 6Y in terms of cellular composition and molecular function (Figures 5B,C,F). The above terms such as GO:0008406, GO: 0007283 and GO: 0007286 are closely related to with gonad and germ cell development, indicating that the genes enriched in these cellular components may play an important role in testis development and spermatogenesis.

KEGG enrichment results showed that the DEcircRNA-derived genes of 0Y vs. 6Y, 0Y vs. 12Y and 0Y vs. 18Y were mainly involved in Autophagy - animal (chx04140), Sphingolipid signaling pathway (chx04071), Longevity regulating pathway (chx04211), AMPK signaling pathway (chx04152), Ras signaling pathway (chx04014), and PI3K-Akt signaling pathway (chx04151) (Figures 5A,D,E); Host genes of DEcircRNA 6Y vs. 12Y, 6Y vs. 18Y and 12Y vs. 18Y were mainly involved in autophagy-animal (chx04140), ubiquitin mediated proteolysis (chx04120), phosphatidylinositol signaling system (chx04070), ErbB signaling pathway (chx04012), and thyroid hormone signaling pathway (chx04919) (Figures 5B,C,F). However, chx04152 (22), chx04014 (23) and chx04151 (24) are signaling pathways closely related to mammalian testicular development and spermatogenesis, suggesting that the differentially expressed genes in these pathways may be involved in the regulation of goat testicular development and spermatogenesis.

### 3.5. Bioinformatics analysis of ceRNA networks

**Figure 6:**
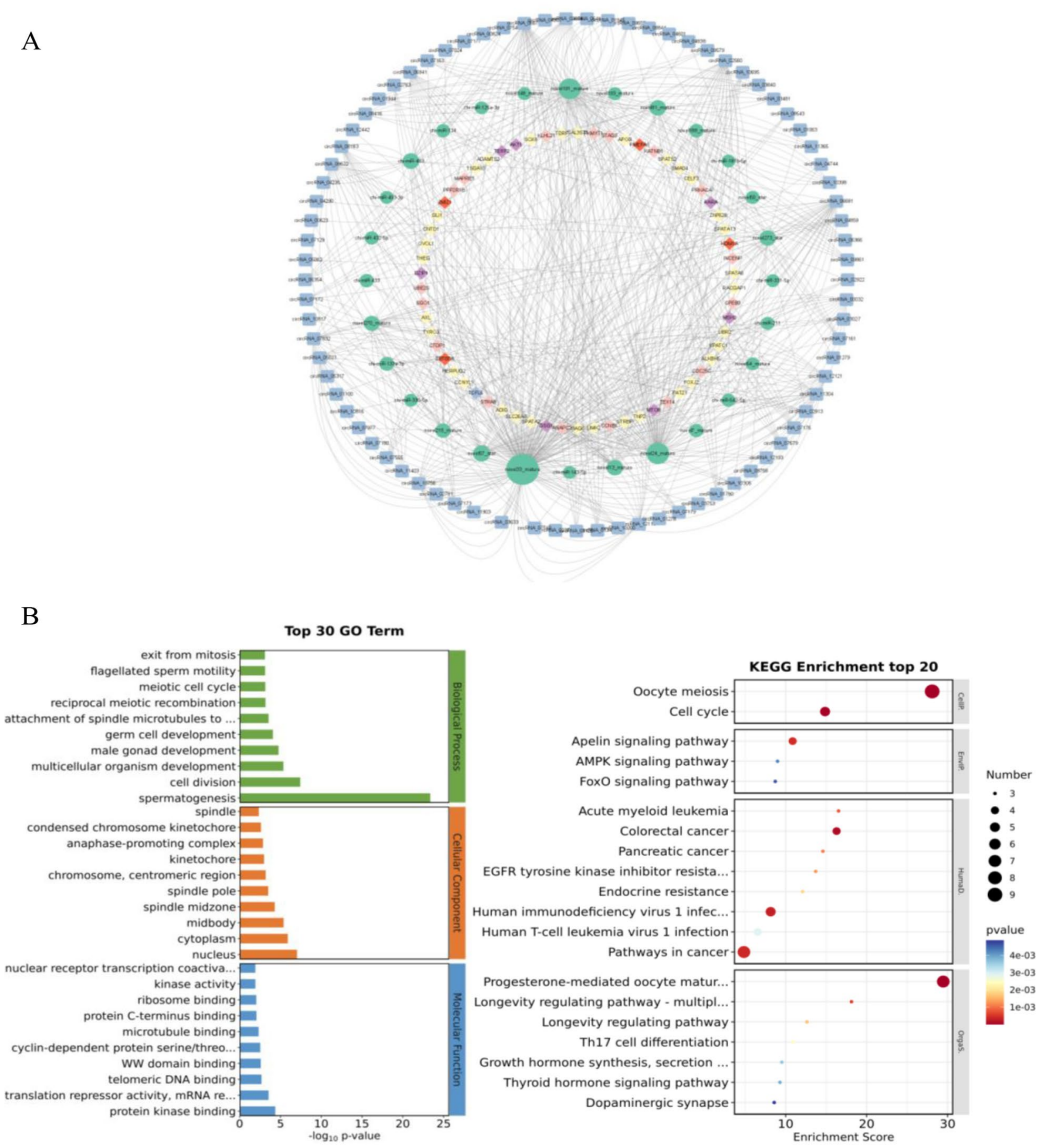
ceRNA network. **(A)** The ceRNA network related to testicular development and spermatogenesis in goats. **(B)** Enrichment analysis of genes in the ceRNA network by GO and KEGG.

ceRNAs are a regulatory mechanism that exists within organisms. Commonly circRNAs compete to bind miRNAs, inhibit their target mRNA translation or cause mRNA degradation, thereby regulating gene expression. Given the important of role circRNAs play in the ceRNA network, we bioinformatically predicted all DEcircRNAs in six comparison groups, yielding a total of 252 circRNA-miRNA relationship pairs and 3,722 miRNA-mRNA relationship pairs. We used 81 highly expressed DEcircRNAs, their corresponding target miRNAs and the mRNAs targeted by these miRNAs, to construct a regulatory network affecting testicular development (Figure 6A). In the ceRNA network, 66 candidate circRNAs, regulating 57 miRNA–mRNA relationship pairs, associated with spermatogenesis, they included 62 mRNAs and 22 miRNAs; 22 candidate were circRNAs were associated with germ cell development, regulating 10 miRNA–mRNA relationship pairs, including 9 mRNAs and 5 miRNAs; 43 were candidate circRNAs associated with the cell cycle, regulating 20 miRNA–mRNA relationship pairs, including 17 mRNAs and 11 miRNAs; and 36 candidate circRNAs associated with androgens, regulating 6 miRNA–mRNA relationship pairs, including 4 mRNAs and 6 miRNAs. Among them, *sox8*, *KATNB1*, *STAG3*, *ZBTB7A* and other mRNAs relevant to testicular development and spermatogenesis were identified. Finally, some important regulatory relationships linked to testicular development and spermatogenesis including were obtained through the ceRNA regulatory mechanism: *circRNA_07172*/ *novel270_mature/sox8*, *circRNA_04859*/*novel270_mature*/ *sox8*, *circRNA_07832*/*chi-miR-433*/*KATNB1*, *circRNA_00032*/*novel24_mature*/*STAG3*, *circRNA_07510*/*chi-miR-133a-3p*/ZBTB7A. We hypothesize that these circRNAs regulate genes associated with testicular development and spermatogenesis by competitive binding to their corresponding miRNAs and play important biological roles in the processes connected with testicular development and spermatogenesis.

The enrichment analysis of relational genes in ceRNA network by GO entry and KEGG pathway showed that kindred genes in the ceRNA network mainly participated in the cell composition (GO: 0005634), cytoplasm (GO: 0005737), chromosome and centromeric region (GO: 0000775). In terms of cell composition, it mainly includes nucleus (GO: 0005634), cytoplasm (GO: 0005737), chromosome and centromeric region (GO: 0000775); Molecular functions mainly focus on protein kinase binding (GO: 0019901,), telomeric DNA binding (GO: 0042162) and microtubule binding (GO: 008017); In terms of spermatogenesis, it mainly includes nucleus (GO: 007283), cell division (GO: 0051301), germ cell development (GO: 007281,) and meiotic cell cycle (GO: 0051321) in biological process. Among them, *sox8*, *KATNB1* and testicular development cognate genes were significantly enriched in GO: 007283 and GO: 0051301 (*p*<0.05) (Figure 6B). These results suggest that these GO functional genes may further regulate the development of goat testes.

KEGG showed that the related genes in the ceRNA network were mainly involved in meiosis (chx04114), cell cycle (chx04110), synthesis, secretion and function of growth hormone (chx04141), AMPK signal pathway (chx04935), and FoxO signal pathway (chx04068). This is consistent with GO entry of the DECircRNA source gene and enrichment analysis of the KEGG pathway (Figure 6B). Among them, chx04935 (25), chx04068 (26) and chx04141 (27) have been identified as regulatory pathways interrelated to testicular development and spermatogenesis. Akt3, the source gene of circRNA_03787 and circRNA_03789 in the chx04935 pathway, is involved in the regulation of SSCS self-renewal, proliferation and differentiation, and is essential for spermatogenesis in male animals (28, 29). In conclusion, based on the function of the source gene, circRNA may play an important role in goat testicular development and spermatogenesis.

## 4. Discussion

The testis is the most important reproductive organ in mammals and its main functions are sperm production and androgen secretion (30). As abundant noncoding RNAs in the eukaryotic transcriptome, circRNAs are important regulators of gene expression and miRNA function at the transcriptional, posttranscriptional and translational levels (31). They are important regulators of gene expression and miRNA function. In recent years, high-throughput RNA sequencing and bioinformatics analysis have revealed that circRNAs located in the cytoplasm can compete with mRNAs for the target binding sites of miRNAs, thereby regulating the expression of mRNAs. For example, 1,432 DEcircRNAs were identified in the spermatozoa of patients with weak spermatozoa, and *circCIT*, *circUSP54*, *circTRMT2B* and *circTADA2A* were identified as marker circRNAs for weak spermatozoa (32). RNA-Seq of Qinchuan cattle at different ages revealed that 2,225 circRNAs were upregulated and 2023 were downregulated in adult cattle testes, and these differentially expressed circRNAs may be involved in testicular development or spermatogenesis in Qinchuan cattle (33). In goats, it was demonstrated that *circP13* significantly promoted differentiation and inhibited apoptosis of primary myogenic cells in goats (34), and *circRN*A8073 indirectly inhibited apoptosis of endometrial epithelial cells, which was beneficial to embryo implantation in goats (35).

In this study, Qianbei Ma goats were selected as the research object. H&E staining and RNA-seq were used to explore the development of the testes of Qianbei Ma goats at different age stages, and key candidate genes and signaling pathways affecting the growth and development of testes of Qianbeima goat were screened. We analyzed the testicular tissue sections of Qianbei Ma goats at different developmental stages and found that the circumference and area of the tubules of the curved sperm increased with age. Sertoli cells in testis increased and Leydig cells decreased, but the number of Sertoli cells and Leydig cells peaked at 6 months of age and maintained a dynamic balance, which was similar to other animal testicular histomorphology studies (36).

We used RNA-seq sequencing technology to further investigate the molecular mechanisms regulating testicular development and spermatogenesis in Qianbei Ma goatS. A total of 12,874 circRNAs were identified from testicular tissues at four different developmental periods. 8,140 differentially expressed circRNAs were detected in six comparison groups of the comparison group, with 4 circRNAs differentially expressed in all four comparison groups.

To explore the role of circRNAs in the testicular development, the source genes of DEcircRNAs were functionally analyzed and GO functional annotation revealed that the source genes of DEcircRNAs were mainly involved in biological processes such as development and reproduction. For example, *circRNA_12624* in 0Y vs. 6Y, 0Y vs. 12Y and 0Y vs. 18Y was derived from the *ATG5* gene. *ATG5* is a cytoplasmic protein mainly expressed in the nucleus, which can be used as a key factor to induce autophagy, regulate cell growth cycle and spermatogenesis of male animals (37–39).The 10 DEcircRNAs in 6Y vs. 12Y, 6Y vs. 18Y and 12Y vs. 18Y were derived from four genes encoding genes associated with germ cell development and spermatogenesis [*PTBP2* (40), *PRMT5* (41), *PLK4*, *SYCE1* (42)]. Of these, *circRNA_04355*, *circRNA_04356* and *circRNA_04357* were all derived from the *PLK4* gene, which has been shown to play a role in the initiation of spermatogenesis (43). Some studies also pointed out that PLK4 mutation is likely to cause male pentaspermia and affect fertility (44). In addition, *circRNA_06790* is produced from the *CTNNB1* gene, which is a key factor in regulating germ cell growth and plays an important role in the stage of differentiation from spermatogonia to spermatocytes to ensure sound fertility in animals (45). In summary, the DEcircRNAs we identified may play important roles in a range of processes related to testicular development, including spermatogenesis, germ cell proliferation, androgen secretion and the cell cycle.

The KEGG analysis of the source genes of DE circRNA showed that in the comparison group of six comparison groups of 0Y vs. 6Y, 0Y vs. 18Y, 6Y vs. 12Y, 12Y vs. 18Y, 0Y vs. 12Y and 6Y vs. 18Y, the Ras, AMPK, Hippo, cAMP, TGF-BETA, PI3K-Akt, and Wnt signaling pathways were enriched in a series of signaling pathways associated with testicular development and spermatogenesis. For example, the Ras signaling pathway participates in the regulation of the CREB family member phosphorylation process, thus promoting the proliferation of spermatogonial stem cells (46); the AMPK/mTOR signaling pathway is involved in regulating autophagy and apoptosis of Sertoli cells. AMPK can also reduce the toxicity of ROS by enhancing the activity of antioxidant enzymes, thus ensuring spermatogenesis in male animals (47). Activation of the YAP1 protein in the Hippo signal pathway can effectively promote the proliferation of testicular germ cells and spermatogenesis (2). The PI3K/Akt signal transduction pathway can promote testosterone secretion through androgen receptor (AR) (48), and the Wnt signal pathway can increase the expression of *StarD7*, and improve the process of testosterone secretion in rat Leydig cells (49). Therefore, the DEcircRNA identified in this study is likely to participate in the biological process of regulating testicular development and spermatogenesis in Qianbei Ma goats.

The ceRNA (competing endogenous RNAs) hypothesis has revealed a new mechanism for endogenous interactions between Rnas. Relevant studies have been conducted in cattle (50), goats (33) and pigs (51). *circBDP1* can regulate the proliferation and differentiation of bovine adipose cells through the *mir-204*/*TRARG1* pathway (50). *circTUT7* influences the development process of porcine embryonic muscle by regulating the expression of HMG20B in the ceRNA mechanism (51). To reveal the functions involved in DEcircRNAs in this study, 81 DEcircRNAs with high expression levels were selected from four aspects of germ cell development, spermatogenesis, androgen and cell cycle, and their corresponding target miRNAs and mRNAs with high scores were predicted. The ceRNA regulatory network affecting testicular development and spermatogenesis was constructed. *sox8* may be regulated by *circRNA_07172*/*novel81_mature* and *circRNA_04859*/*novel270_mature* pairs in the network. *novel81_mature* and *novel270_mature* are novel miRNAs identified in this study, and there are no related studies. *sox8* is a product of adult Sertoli cells. It is a regulatory component of the testicular barrier and a key factor in maintaining normal fertility in male animals (52). Its elimination not only leads to the disorder of the spermatogenic cycle, but also causes abnormal motility of sperm in the epididymis, which affects the reproductive ability of animals (53). *STAG3*, a protein encoding meiotic condensation complex, can regulate germ cell development and is a target gene of the circRNA_00032/novel24_mature regulatory axis in the network (54). Previous studies have found that *STAG3* gene mutations can cause male infertility due to meiotic arrest, and *STAG3* knockout can cause azoospermia in male mice (55, 56). *Chi-miR-433* in the regulatory axis of *circRNA_07832/chi-miR-433*/*KATNB1* has been confirmed to participate in animal growth and development processes such as goat hair follicle development and muscle differentiation, but its role in testicular development and spermatogenesis has rarely been studied (57, 58). In this study, the target gene *KATNB1* regulated by *circRNA_07832*/*chi-miR-433* is a key molecule involved in testicular development. It can regulate the growth, development and reproduction of mammals by regulating the cycle of fibroblasts and spermatocytes. (59). *ZBTB7A* is a transcription inhibitor belonging to the POK (POZ/BTB and Kruppel) protein family (60). Studies have shown that its family proteins *ZBTB42* and *ZBTB40* participate in biological processes such as animal germ cell development and sperm maturation (61, 62). In this study, *ZBTB7A* was regulated by the *circRNA_07510*/*chi-miR-133a-3p* relationship pair. Therefore, *circRNA_07172*, *circRNA_04859*, *circRNA_00032*, *circRNA_07832*, and *circRNA_07510* are closely related to testicular development and spermatogenesis. They may inhibit the activity of miRNA and promote the expression of mRNA by binding to miRNA response elements (MREs), thereby affecting the biological process of testicular development and spermatogenesis, but the mechanism needs to be further studied.

After functional enrichment analysis of the relational genes in the ceRNA Network, GO entry analysis showed that the relevant genes were distributed to several major biological processes, including spermatogenesis, testicular development, cellular processes and reproduction. This was consistent with the enrichment entry of the source gene GO. The enrichment results of the KEGG pathway analysis showed that the corresponding genes were mainly enriched in the MAPK signaling pathway, cAMP signaling pathway, Wnt signaling pathway, Ras signaling pathway, and PI3K-Akt signaling pathways, which are related to testicular development and spermatogenesis. Studies have shown that the WNT signaling pathway can induce morphological changes and cell movement in mouse and human spermatogonia through its main member WNT3A triggering membrane extension (63). The cAMP signaling pathway can regulate the proliferation of sertoli cells through tight connections and adhesive connections, and Sertoli cells participate in the formation of the testicular barrier to ensure the stability of microenvironment in seminiferous tubules and promote spermatogenesis (47). Many studies have also demonstrated that the PI3K-Akt signaling pathway plays an important role in testicular development and spermatogenesis in animals (64). For example, Meroni S et al. detected that FSH positively regulates the proliferation of Sertoli cells by activating the PI3K-dependent pathway to increase the transcriptional activity of HIF 2 (hypoxia-inducible factor) and cyclin D1 expression, thus promoting spermatogenesis (65).Therefore, the above analysis indicates that the genes in the ceRNA network are inextricably linked to testicular development and spermatogenesis, and these circRNAs also play an important role in testicular development and spermatogenesis in Qianbei Ma goats, but their specific regulatory mechanisms need to be further investigated.

## 5. Conclusion

In this study, we found that the circumference and area of seminiferous tubules and the number of sperm in the testis of Qianbei Ma goats increased with age. A total of 12,784 circRNAs were identified in the testis of Qianbei Ma goats, and 8,140 DEcircRNAs were obtained from six comparison groups(0Yvs6Y, 6Yvs12Y, 12Yvs18Y, 0Yvs18Y, 0Y vs. 12Y, 6Y vs. 18Y). The genes of origin of these DEcircRNAs are mainly involved in spermatogenesis and testicular development. *circRNA_07172*, *circRNA_04859*, *circRNA_00032*, *circRNA_07832* and *circRNA_07510* may play key roles in testicular development and spermatogenesis in goats. This study enriches the existing database of circRNAs in goat testis development and spermatogenesis.

For additional requirements for specific article types and further information please refer to “Article types” on every Frontiers journal page.

## Data availability statement

The datasets presented in this study can be found in online repositories. The names of the repository/repositories and accession number(s) can be found at: https://www.ncbi.nlm.nih.gov/bioproject/PRJNA917820.

## Ethics statement

The studies involving animals have been reviewed and approved by the Animal Protection and Use Committee of Guizhou University, Guiyang, China (Approval number: EAE-GZU-2021-E021). The animal handling procedures were in line with the Chinese Animal Welfare Guidelines and were approved by the Animal Protection and Use Committee of Guizhou University, Guiyang, China (Approval number: EAE-GZU-2021-E021).

## Author contributions

WT and XC conceived and designed the experiments. WT, KF, WG, and ZA conducted the experiments. WT and WG analyzed the data. WT wrote the paper. XC, WG, and ZA revised the paper. All authors contributed to the article and approved the submitted version.

## Funding

This research was funded by the National Natural Science Foundation of China (32260835), the Science and Technology Project of Guizhou Province [Qian Kehe Foundation—ZK (2021) General 151], and the Guizhou High-Level Innovative Talents Project [Qian Kehe Platform Talents (2022) 021–1].

## Conflict of interest

The authors declare that the research was conducted in the absence of any commercial or financial relationships that could be construed as a potential conflict of interest.

## Publisher’s note

All claims expressed in this article are solely those of the authors and do not necessarily represent those of their affiliated organizations, or those of the publisher, the editors and the reviewers. Any product that may be evaluated in this article, or claim that may be made by its manufacturer, is not guaranteed or endorsed by the publisher.

## Footnotes

^1^ https://www.ncbi.nlm.nih.gov/genome/?term=Capra+hircus

^2^ http://kobas.cbi.pku.edu.cn/

^3^ http://www.microrna.org/microrna/home.do

^4^ http://www.circbank.cn/

^5^ http://www.targetscan.org

^6^ http://www.mirdb.org/
